# Receptor tyrosine kinases: biological functions and anticancer targeted therapy

**DOI:** 10.1002/mco2.446

**Published:** 2023-12-07

**Authors:** Nan Zhang, Yongsheng Li

**Affiliations:** ^1^ Chongqing University Cancer Hospital, School of Medicine Chongqing University Chongqing China; ^2^ Department of Medical Oncology Chongqing University Cancer Hospital Chongqing China

**Keywords:** classification, gene mutation, receptor activation, receptor tyrosine kinases, targeted therapy

## Abstract

Receptor tyrosine kinases (RTKs) are a class of protein kinases that play crucial roles in various cellular processes, including cell migration, morphological differentiation, cell growth, and angiogenesis. In humans, 58 RTKs have been identified and categorized into 20 distinct families based on the composition of their extracellular regions. RTKs are primarily activated by specific ligands that bind to their extracellular region. They not only regulate tumor transformation, proliferation, metastasis, drug resistance, and angiogenesis, but also initiate and maintain the self‐renewal and cloning ability of cancer stem cells. Accurate diagnosis and grading of tumors with dysregulated RTKs are essential in clinical practice. There is a growing body of evidence supporting the benefits of RTKs‐targeted therapies for cancer patients, and researchers are actively exploring new targets and developing targeted agents. However, further optimization of RTK inhibitors is necessary to effectively target the diverse RTK alterations observed in human cancers. This review provides insights into the classification, structure, activation mechanisms, and expression of RTKs in tumors. It also highlights the research advances in RTKs targeted anticancer therapy and emphasizes their significance in optimizing cancer diagnosis and treatment strategies.

## Graphical abstract

RTKs play a crucial role in the development of cancer. RTKs affect transcription‐factor activation and histone/chromatin modification by activating downstream pathways such as MAPK/ERK and PI3K/AKT, which in turn cause proliferation, resistance, metastasis, angiogenesis, and invasion of tumors.

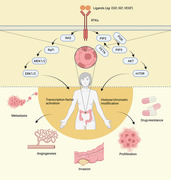

## 1 INTRODUCTION

It has been increasingly recognized that the fundamental mechanism behind cellular carcinogenesis is the uncontrolled proliferation of cells due to the disruption of cellular signal transduction pathways. This disruption is a result of the rapid advancements in tumor biology and related sciences. Both external and internal signals, such as hormones, neurotransmitters, cytokines, and temperature, interact with cells and trigger various cellular effects through multiple pathways. The process through which these diverse cellular signals travel across cell membranes and signaling molecules to modify cellular gene expression is referred to as signal transduction.^1^ Intracellular signaling is believed to play a crucial role in almost all significant biological phenomena. Abnormalities in cellular signaling mechanisms that impact cell growth, differentiation, metabolism, and overall behavior can lead to various diseases, including cancer.^2^, ^3^, ^4^, ^5^

The signaling process involves several factors, such as adenylate cyclase, phospholipase C, protein kinase A, protein kinase C, serine/threonine kinase, G proteins, adenosine 5′‐triphosphate (ATP), and calcium.^1^, ^6^ Among these factors, receptor tyrosine kinases (RTKs) play a crucial role in cellular signaling and have various physiological activities. RTKs control important functions in tissue homeostasis, cell function, and mammalian development. These functions include tissue repair and regeneration, organ morphogenesis, cell proliferation and survival, and neovascularization.^7^, ^8^, ^9^, ^10^, ^11^ Therefore, gaining a deeper understanding of RTKs can help identify potential targets for cancer detection and therapy.

This review focuses on the classification and structure of RTKs, the mechanisms of normal and oncogenic activation, their expression in high‐incidence tumors, and the different types of targeted drugs. The aim is to contribute to cutting‐edge research and improve understandings on clinical diagnosis and treatment related to RTKs.

## 2 CLASSIFICATION OF RTKs

RTKs are a specific type of tyrosine kinases that play a crucial role in facilitating intercellular communication and regulating various intricate biological processes such as cell growth, motility, differentiation, and metabolism. The 58 RTKs identified in humans are categorized into following 20 distinct families based primarily on the composition of their extracellular regions (Table 1).

**TABLE 1:** RTK subfamily classification based on kinase domain sequence.

Class	Family	Receptors	Number
I	EGF/ErbB	EGFR, ErbB2/HER2, ErbB3/HER3, ErbB4/HER3	4
II	Ins	InsR, IGF1R, InsRR	3
III	PDGF	PDGFRα, PDGFRβ, CSF1R, KIT, FLT3	5
IV	VEGF	VEGFR1/Flt1, VEGFR2/KDR, VEGFR3/Flt4	3
V	FGF	FGFR1, FGFR2, FGFR3, FGER4	4
VI	PKT7	PKT7/CCK4	1
VII	TRK	TRKA, TRKB, TRKC	3
VIII	ROR	ROR1, ROR2	2
IX	MuSK	MuSK	1
X	HGF	MET, MST1R (RON)	2
XI	TAM	AXL, MER, TYRO3	3
XII	TIE	TIE1, TEK (TIE2)	2
XIII	Eph	EphA1‐8, EphA10, EphB1‐4, EphB6	14
XIV	RET	RET	1
XV	RYK	RYK	1
XVI	DDR	DDR1, DDR2	2
XVII	ROS	ROS	1
XVIII	LMR	LMR1, LMR2, LMR3	3
XIX	ALK	LTK, ALK	2
XX	STYK1	STYK1	1

The epidermal growth factor (EGF/ErbB) receptor family comprises several main members, including EGFR (HER1/Erb1), HER2 (Erb2), HER3 (Erb3), and HER4 (Erb4) receptors.^12^ These human epidermal growth factor receptors (HERs) are frequently observed to be highly expressed in epithelial cell tumors, such as colorectal cancer (CRC),^13^ head and neck squamous cell carcinoma,^14^ non‐small cell lung cancer (NSCLC),^15^, ^16^ breast cancer,^17^ pancreatic cancer,^18^ and renal cell carcinoma (RCC).^19^ The insulin growth factor/insulin receptor family (IGFR/InsR) consists of IGFR and IRR receptors. Both IGF1 and IGF2 can bind to and activate the IGF1R transmembrane receptor kinase. However, when IGF2 binds, it does not activate any downstream signaling pathway due to the absence of a kinase structural domain in IGF2R.^20^

Platelet‐derived growth factor receptor (PDGFR), colony‐stimulating factor 1 receptor (CSF1R), KIT proto‐oncogene RTK (KIT), and FMS‐related tyrosine kinase 3 (FLT3) receptors play crucial roles in various cellular processes. PDGF is a vital growth factor responsible for healthy tissue growth and division, as well as contributing to the formation of blood vessels. On the other hand, CSF‐1R, which is secreted by cancer cells into the tumor environment as a strategy to evade the immune system, promotes the growth and recruitment of immunosuppressive myeloid cells. Consequently, the presence of CSF‐1R‐expressing myeloid cells within the tumor is associated with decreased survival rates in many malignancies.^21^ The vascular endothelial growth factor (VEGF) receptor family, which consists of VEGFR‐1, VEGFR‐2, and VEGFR‐3 receptors, plays a crucial role in regulating various physiological processes including metabolic homeostasis, cell migration, proliferation, angiogenesis, and lymphangiogenesis.^22^, ^23^ Similarly, the fibroblast growth factor (FGF) receptor family, comprising FGFR1, FGFR2, FGFR3, and FGFR4 receptors, is involved in mediating metabolic processes, tissue repair, adult tissue regeneration, as well as growth, differentiation, survival, and patterning of progenitor cells during embryonic development and organogenesis.^24^, ^25^

Protein tyrosine kinase‐like 7 (PTK7) and colon carcinoma Kinase 4 (CCK4) receptors are involved in the polarization of epithelial cells and the formation of brain structures. While the gene product is found to be catalytically active as a protein kinase through sequence analysis, it is also known to play a role in the Wnt^26^ and VEGF^27^ signaling pathways.

Neurotrophin receptor/tropomyosin receptor kinase (TRK, NTRK) family comprises of TRKA, TRKB, and TRKC receptors. These receptors play a crucial role in the proliferation and migration processes of the nervous system. Specifically, TRKA, TRKB, and TRKC receptors respond to nerve growth factor (NGF), brain‐derived neurotrophic factor (BDNF), and neurotrophin‐3, respectively.^22^

The RTK‐like orphan receptor (ROR) family consists of ROR1 and ROR2 receptors. ROR1 acts as a substitute receptor and co‐receptor 1 for Wnt signaling, controlling cell division, polarity, and tissue maintenance.^28^ On the other hand, the role of ROR2 in tumor development depends on the type or stage of the tumor. It can function as an atypical Wnt signaling agent, either repressing or activating the tumor.^29^

The muscle‐specific kinase (MuSK) receptor plays a crucial role in the formation and organization of neuromuscular junctions in skeletal muscle.^30^ The hepatocyte growth factor (HGF) receptor family consists of mesenchymal‐epithelial transition factor (MET) and recepteur d′origine nantais (RON) receptors. When HGF binds to its receptor MET (c‐Met), it triggers the proliferation, migration, and morphogenesis of epithelial cells.^31^, ^32^

The TAM (TYRO3, AXL, and MER) receptors are activated by the vitamin K‐dependent proteins growth arrest‐specific protein 6 (Gas6) and protein S. The activation of TAM receptors by these proteins influences various cellular processes including cell proliferation, survival, adhesion, and migration.^33^ TAM receptors have been found to have anti‐inflammatory properties and have been implicated in carcinogenesis in several malignancies.^34^

Tyrosine kinase with Immunoglobulin‐like and EGF‐like domains (TIE) is a receptor family consisting of TIE1 and TIE2 receptors. These receptors play a crucial role in regulating angiogenic and lymphangiogenic responses.^35^ The Ephrin (Eph) receptor family includes EphA1, EphA2, EphA3, EphA4, EphA5, EphA6, EphA7, EphA8, EphA10, EphB1, EphB2, EphB3, EphB4, and EphB6 receptors. Eph receptors are involved in controlling angiogenesis, cell migration, patterning, and the formation of neuronal cells.^36^, ^37^, ^38^ The activation of the rearranged during transfection (RET) receptor by ligands of the glial cell‐derived neurotrophic factor family plays a crucial role in cell proliferation, neuronal navigation, cell migration, and cell differentiation.^39^

The related to tyrosine kinase (RYK) receptor is characterized by functional extracellular Wnt‐binding domains and is closely linked to Wnt signaling.^40^ The discoidin domain receptor (DDR) family comprises DDR1 and DDR2. Activation of DDR1 plays a crucial role in regulating cell adhesion, proliferation, and metalloproteinase expression.^41^ DDR1 promotes tumor cell invasion and enhances the survival of tumor stem cells in a collagen‐rich environment.^42^

The presence of the reactive oxygen species (ROS) receptor family has been observed in various malignant tumors, suggesting that this protein could be a promising target for anticancer drugs.^43^, ^44^ Lemur receptor kinases (LMR/LMTK) are known to be associated with cancer and have an impact on multiple signaling pathways involved in cell proliferation, migration, and invasiveness.^45^

The members of the anaplastic lymphoma kinase (ALK) receptor family consist of ALK and leukocyte tyrosine kinase (LTK). The fusion of the ALK genome is responsible for the formation of numerous tumors.^46^ Additionally, the serine/threonine/tyrosine kinase (STYK) receptor plays a role in various cellular and developmental processes, including cell proliferation, differentiation, and survival.^47^, ^48^

## 3 STRUCTURE AND ACTIVATION MECHANISMS OF RTKs

The structure of RTKs consists of an extracellular ligand binding domain, one transmembrane helix, and an intracellular region comprising a juxtamembrane regulatory region, a tyrosine kinase domain (TKD), and a carboxyl (C‐) terminal tail.^49^ RTKs are primarily activated by receptor‐specific ligands, such as growth factors, which bind to the extracellular region of the receptor. This binding leads to receptor dimerization and/or oligomerization, resulting in receptor activation.^50^ Upon activation, each TKD undergoes trans autophosphorylation, and the cis autoinhibition is released due to conformational changes.^50^ As a result, the TKD adopts an active conformation. Furthermore, RTK autophosphorylation attracts and activates downstream signaling proteins, which play crucial roles in various physiological signaling pathways.^51^ We introduce common activation mechanisms below.

### 3.1 Ligand‐induced dimerization of RTKs

The preformed dimer of the receptor can exist in either an “inactive” or “active” state, as the receptor maintains a dynamic balance between the dimer and the monomer. It is likely that the “inactive” and “active” dimers are in a state of dynamic equilibrium. When a ligand binds to the receptor, it can alter this equilibrium and induce dimerization.^52^, ^53^, ^54^ There are generally four modes of RTK dimerization that lead to the activation of tyrosine kinase structural domains. These modes can be categorized into two mechanical extremes and two intermediate cases. In one extreme, exemplified by TrkA, receptor dimerization is solely mediated by the ligand and does not involve any direct contact between the two receptors.^55^ On the other extreme, dimerization is entirely mediated by the receptors themselves, without any physical interaction between the two activating ligands, as seen in ErbB family members.^56^ An intermediate situation occurs when a ligand homodimer binds to two receptor molecules before interacting with them through the dimer interface, as observed in the case of KIT.^57^ Another intermediate circumstance arises when an auxiliary molecule not only binds bivalent ligands and makes direct receptor‐receptor interactions but also participates in receptor dimerization. For example, the FGFR family utilizes heparin or heparan sulfate as an auxiliary molecule in this mode.^58^, ^59^

### 3.2 Activation of intracellular TKDs

Prior to activation, TKD is inhibited in a *cis* conformation through specific intramolecular interactions.^60^, ^61^ This *cis* autoinhibition can be relieved by ligand‐induced dimerization. Each TKD's receptor has distinct intramolecular interactions that contribute to cis autoinhibition. The pivotal step in RTK activation occurs when the cis autoinhibition is released upon ligand‐induced receptor dimerization.

The self‐inhibition of RTKs can be observed through the activation loop structure of the insulin receptor TKD.^62^ In this study, a novel finding is presented, demonstrating that the activation loop structure of the insulin receptor TKD is connected to a crucial tyrosine (Y1162) in the kinase. This connection serves to stabilize the activation loop structure, blocking the active site and impeding access to ATP and protein substrates. Consequently, the insulin receptor TKD is self‐inhibited in cis through its own activation loop. However, when the receptor is engaged, trans‐phosphorylation disrupts the cis‐autoinhibitory connections, leading to the relaxation of the insulin receptor's TKD into an activated state. This process is achieved through autophosphorylation, which effectively “releases” the cis‐autoinhibition. It is worth noting that activation loop autoinhibition also impacts other receptors, such as FGFR and IGF‐1R, in addition to the insulin receptor.^63^, ^64^

Juxtamembrane domain autoinhibition is an additional regulatory mechanism that complements the activation loop. The juxtamembrane domain sequences play a crucial role in interacting with various sections of the TKD, thereby stabilizing an autoinhibited conformation. This mechanism is responsible for controlling KIT and Eph receptors.^61^, ^65^ The self‐inhibitory connections can be disrupted by phosphorylating specific tyrosine residues in the juxtamembrane domain, leading to the adoption of an active conformation by TKD.^62^

C‐terminal tail inhibition refers to the process where the C‐terminal tail of receptors such as TEK, MET, and RON (MST1R) interacts with the active site of TKD, preventing the entry of substrates.^60^ In the case of Tie2, the nucleotide‐binding loop adopts an inactive conformation, and the C‐terminal tail region containing the tyrosine autophosphorylation site hinders substrate access to the active site. This interaction stabilizes the inactive conformation and effectively inhibits the kinase activity. However, when ligand‐induced dimerization occurs, the kinase adopts an active configuration by undergoing trans‐phosphorylation of important tyrosine residues. This process disrupts the self‐inhibitory connections, leading to the activation of the kinase.

### 3.3 Mechanism of activation of downstream signaling

**FIGURE 1:**
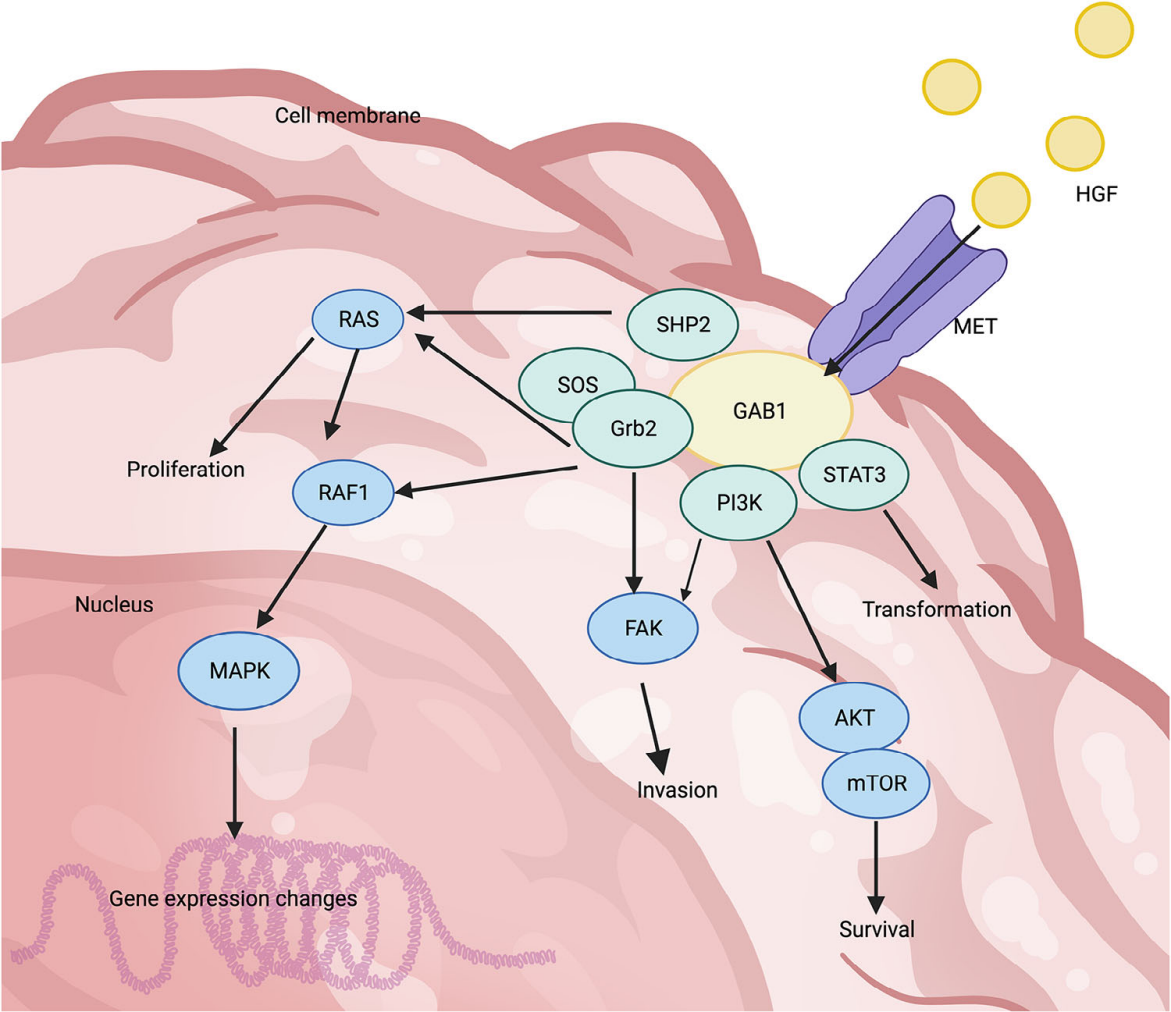
The HGF/MET signaling pathway. Following specific binding of HGF to the MET receptor in the extracellular domain, activated MET has the ability to recruit and phosphorylate various effector proteins. These include growth factor receptor binding protein (Grb2), transcriptional activator STAT3, as well as other substrate and junctional proteins. Furthermore, activated GAB1 serves as a binding site for downstream proteins such as SHP2 and PI3K, thereby influencing the survival, proliferation, invasion, and gene expression of tumor cells through signaling pathways like RAS–MAPK and PI3K–AKT. Created with BioRender.com.

The activation of RTK and subsequent autophosphorylation of this protein leads to the completion of various downstream signaling proteins. The autophosphorylation event in “phase I” primarily serves to enhance the catalytic activity of the kinase after the receptor binds to its activating ligand. The “phase II” autophosphorylation events require prior activation of the kinase in “phase I” and the formation of a binding site based on phosphotyrosine. This binding site recruits cytoplasmic signaling molecules that contain Src homology‐2 (SH2) and phosphotyrosine‐binding (PTB) domains.^50^, ^66^ Upon recognition of both types of proteins, RTK functions as a kinase, transferring its own phosphate to both proteins and leading to subsequent intracellular effects. The activated RTK has the ability to attract and regulate multiple signaling pathways due to the presence of numerous phosphotyrosines and the involvement of various docking proteins. For instance, in the case of MET, these downstream signaling pathways include important ones such as RAS/MAPK, PI3K/AKT, and STAT3 (Figure 1).^67^ Consequently, RTK activates transcriptional pathways that play a role in controlling various cellular activities and acts as a central hub for transmitting complex information about cell growth and migration from the extracellular environment to the nucleus.

## 4 MECHANISM OF ONCOGENIC ACTIVATION OF RTKs

**FIGURE 2:**
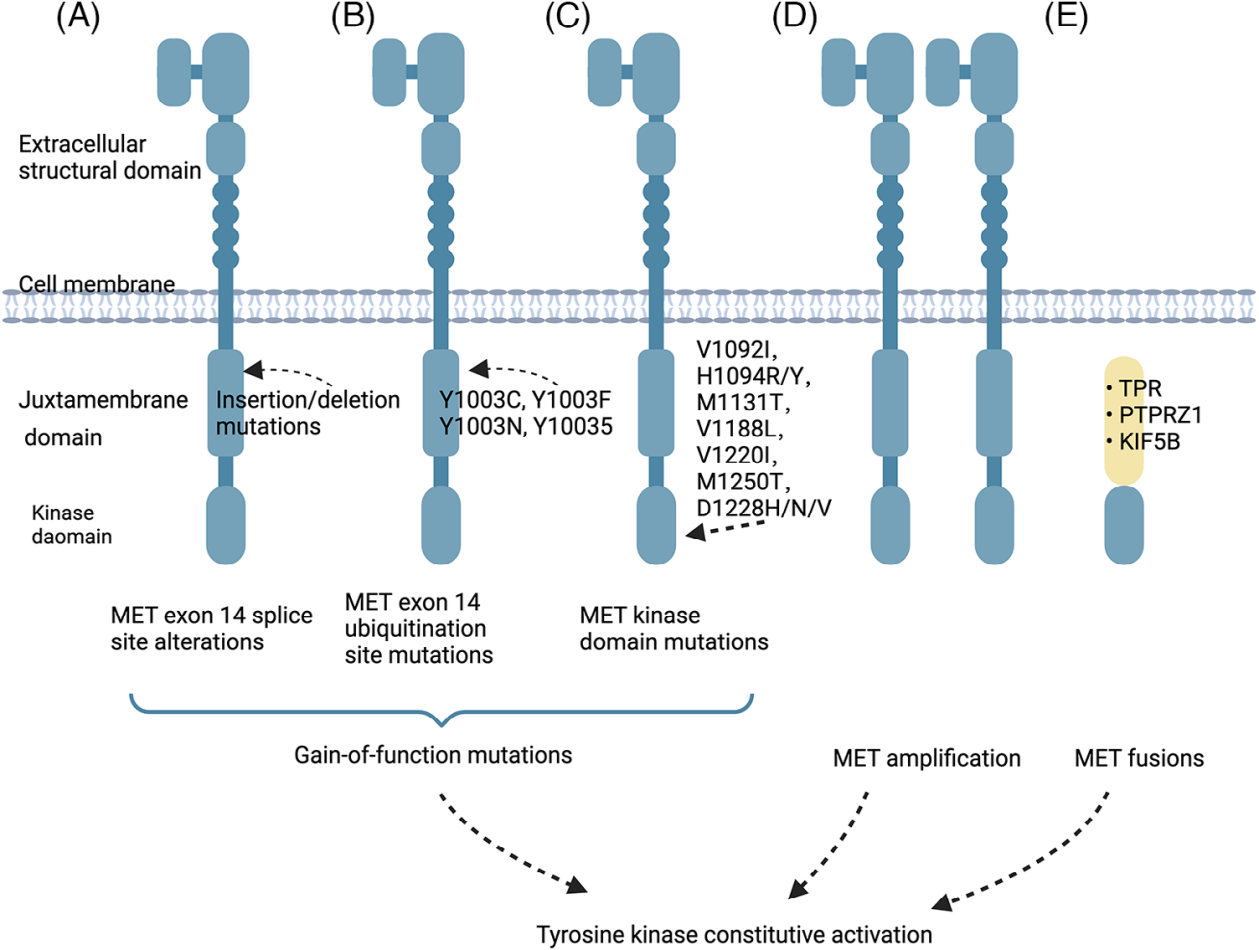
Genetic material alterations leading to MET activation in human cancers. (A) The exclusion of Exon 14 is a consequence of the MET exon 14 splice site mutation. These splice variants lead to the breakdown of MET and an increase in MET signaling due to the absence of a ubiquitin‐binding site in the juxtamembrane region. (B) Missense mutations that affect the ubiquitylation location of the MET protein's Y1003 or the region of the MET pre‐mRNA responsible for coding the juxtamembrane domain hinder or modify the binding of spliceosomes. Ultimately, these mutations replicate the biological effects of alterations to the MET exon 14 splice site. (C) Mutations in the structural domain of the kinase lead to increased activation of MET kinase. (D) Focal MET amplification is responsible for producing higher levels of MET transcription and expression. This amplification also leads to increased ligand‐independent oncogenic signaling through receptor autodimerization or oligomerization, as well as autophosphorylation. (E) The gene rearrangements involving the MET TKD result in the formation of fusion proteins. Created with BioRender.com.

Constitutive activation of RTKs can confer oncogenic characteristics to normal cells and initiate RTK‐induced carcinogenesis.^68^ The primary mechanisms responsible for RTK activation in human cancers include three modes of genetic alterations: gain‐of‐function mutations, overexpression and genomic amplification, and chromosomal rearrangements (Figure 2). Autocrine activation also contributes to this process.^69^ Furthermore, other factors such as the tumor microenvironment (TME) and the maintenance of tumor stem cells (CSCs) play a role.

### 4.1 Gain‐of‐function mutations

Acquired functional mutations have altered the downstream signaling of the RTK gene, resulting in its suppression no longer being the norm. Mutations in proto‐oncogenes can provide cells with a selective growth advantage. These driver genes not only offer the potential for targeted treatment of malignancies but also contribute to our understanding of the mechanisms involved in carcinogenesis and development. Somatic mutations in genes encoding RTKs often occur in evolutionarily conserved residues, such as the kinase activation loop and the DFG motif surrounding the nucleotide binding pocket. These conserved residues (D, F, and G) play crucial roles in ATP binding and catalytic activity.^70^, ^71^

Notable examples of the mutation spectrum in RTKs can be observed in *MET* somatic cell mutations. Variants within the *MET* exon 14 region consist of various nonhomologous mutations that can lead to the activation and perpetuation of the MET pathway. Among these mutations, base substitution and insertion loss are equally prevalent and significant contributors to the development of cancer.^72^, ^73^ Upon activation of MET protein, its Y1003 site is typically phosphorylated.^74^ This phosphorylation event enables MET to bind with c‐Cbl E3 ligase, resulting in the ubiquitination modification of MET protein. The modified protein is then degraded, thus forming a self‐regulated negative feedback loop.^75^ The skipping of *MET* exon 14 results in the loss of ubiquitination of MET proteins due to mutations in its receptor binding site. This deletion causes the removal of the E3 ubiquitin ligase c‐Cbl, which in turn increases the instability of MET proteins. As a result, downstream signaling pathways are activated, leading to tumorigenesis. Although *MET* exon 14 alterations were originally identified in small cell lung cancers (SCLCs),^76^ subsequent studies have found that these genetic variants are more common in NSCLC, accounting for approximately 3−4% of cases.^72^, ^73^, ^77^, ^78^

Mutations can also occur in the extracellular structural domain, transmembrane structural domain, and near‐membrane structural domain of RTK. These mutations are linked to an elevated expression of RTKs, resulting in their phosphorylation even in the absence of ligand stimulation.^79^ In 1997, mutations in the structural domain of *MET* kinase were first reported in hereditary papillary renal carcinoma.^80^ These mutations, which include V1092I, H1094R/Y, M1131T, V1188L, V1220I, M1250T, and D1228H/N/V, have been shown to increase kinase activity and may modify phenotype or form tumor foci.^81^ These mutations have also been found to induce tumorigenesis in mouse models.^81^, ^82^ Twenty years later, somatic *MET* kinase mutations, including V1092I, H1094L/R/Y, N1100Y, H1106D, M1131T, V1188L, L1195V, V1220I, D1228H/N/V, Y1230A/C/D/H, Y1235D, and M1250I/T, in the structural domain were found in sporadic papillary RCC (PRCC).^80^, ^83^, ^84^ These mutations, which were accompanied by *MET* amplification, showed lower activity. Similarly, mutations in the structural domain of *MET* kinase have also been observed in other tumors like hepatocellular carcinoma (HCC) and head and neck tumors.^82^, ^85^, ^86^

### 4.2 Overexpression and genomic amplification

RTKs are frequently found to be overexpressed in various types of human cancers. This overexpression leads to an increased concentration of the receptor in the affected area, which in turn results in the activation of RTKs signaling pathways. These pathways play a crucial role in counteracting antagonistic regulation.^87^ There are two ways in which an increase in *MET* copy number can occur: through multimerization or amplification. Polymorphism arises when the replication of chromosome 7, where *MET* is located, is not properly carried out.^88^, ^89^ Polyploidy cannot be considered as a driver gene. Instead, *MET* amplification is defined as a local acquisition with local gene duplication. This type of amplification can effectively enhance the *MET* copy number without replicating chromosome 7.^90^ Therefore, compared with the broad increase on chromosome 7, the focused *MET* amplification more accurately represents the real oncogenic driving state.^91^ Preclinical studies have shown that *MET* amplification can lead to upregulated *MET* expression, ligand‐independent receptor activation, and downstream signaling.^92^, ^93^

Gene amplification is the main mechanism that leads to the overexpression of RTKs. However, RTKs can also be overexpressed through transcriptional/translational enhancement,^94^ oncogenic viruses,^95^ derailments of normal regulatory mechanisms such as loss of phosphatases^96^  or other negative regulatory factors.^97^, ^98^

### 4.3 Chromosomal rearrangements

New tyrosine kinase fusion oncoproteins are formed due to various chromosomal rearrangements.^78^, ^99^, ^100^ As a result, small molecule inhibitors are often developed to target these abnormal fusion proteins for therapeutic purposes. The first tyrosine kinase fusion to be identified was BCR–ABL. This fusion resulted from the translocation t (9,22), also known as the “Philadelphia chromosome”. It involved the joining of the *ABL1* tyrosine kinase genes on chromosome 9 with the BCR gene on chromosome 22, creating the *BCR–ABL* fusion oncoprotein.^101^ Patients with chronic myelogano leukemia (CML) and certain patients with acute lymphoblastic leukemia are known to have *BCR–ABL* fusion.^102^, ^103^ Subsequently, the introduction of imatinib, a medication that targets the ABL kinase, revolutionized the treatment of CML patients and marked the beginning of targeted therapies.^104^, ^105^

While *BCR–ABL* is primarily observed in leukemia, numerous tyrosine kinase fusions have been identified in various types of tumors, encompassing both liquid and solid malignancies. For example, MET fusions have been identified in various types of cancer such as osteosarcoma,^106^ gastric cancer,^107^ thyroid cancer,^108^ lung cancer,^109^ glioma,^110^ and sarcoma.^111^ In 1984, the MET gene was first identified in a chemically induced human osteoma cell line as part of an oncogenic fusion with the translocated promoter region (*TPR*) gene.^112^ Apart from *TPR–MET*, which was initially discovered in osteosarcoma.^112^ numerous other *MET* fusions have also been documented.^99^, ^113^, ^114^, ^115^, ^116^, ^117^, ^118^, ^119^, ^120^, ^121^ *MET* fusions can be achieved by either interchromosomal fusions, such as *KIF5B–MET*, or intrachromosomal fusions, such as *PTPRZ–MET*. These fusions typically consist of the kinase region, which is encoded by exon 15 of the *MET* gene, and the dimerized regions of various upstream partner genes.^99^, ^113^, ^115^, ^116^, ^120^, ^122^, ^123^, ^124^ This leads to an activation of *MET* substitution that is independent of ligands. The *PTPRZ* promoter is commonly associated with the complete *MET* gene in the *PTPRZ–MET* fusion, resulting in elevated expression of MET and activation of its downstream signaling pathways.^122^, ^125^

### 4.4 Autocrine activation

Growth factors and cytokines serve as messengers in cell‐cell communication, transmitting signals from the secreting cell to a distant target cell. When the target cell secretes the cell itself, it is referred to as “autocrine.” Constitutive autocrine activation can lead to clonal growth and tumor development. In various malignancies, autocrine activation of several RTKs such as TGFα‐EGFR,^126^ HGF‐MET,^127^, ^128^ and SCF‐KIT^129^, ^130^, ^131^ is well documented. Taking the HGF‐MET autocrine loop as an example, sustained activation of multiple MET proteins occurs in a ligand‐dependent manner.^132^ HGF‐producing and expressing cells engage in crosstalk within the TME, including between the stromal and epithelial components, as well as among stromal cells like tumor‐associated macrophages and neutrophils.^133^ It is noteworthy that many stimuli that trigger MET transcription in cancer cells also lead to HGF upregulation in the tumor stroma, creating a feedforward stimulatory circuit that enhances MET activation.^127^, ^128^ Autocrine mechanisms can be a promising target for cancer therapy.^126^ One example is the case of EGFR‐mutated lung cancer cells, which show reduced responsiveness to EGFR‐tyrosine kinase inhibitor (TKI) due to the presence of a ligand/receptor autocrine loop.^134^

### 4.5 Alterations in the TME

The TME refers to the internal environment in which tumor cells develop and exist. It plays a crucial role in the development of cancer.^135^ One of the important cellular components of the TME is macrophages. AXL, a protein, contributes to immune suppression and the development of a preneoplastic phenotype, which is highly expressed in macrophages associated with tumors.^136^ The protein HGF is primarily secreted by cancer‐associated fibroblasts (CAFs) and has a paracrine effect on nearby tumor cells.^137^, ^138^ When HGF binds to MET, it triggers the auto‐phosphorylation of tyrosine residues (Tyr1234, Tyr1235) in the cytoplasm. This, in turn, activates the intracytoplasmic protein tyrosine kinase (PTK) structural domain of c‐Met, leading to the phosphorylation of c‐Met C‐terminal tyrosine (Tyr1349, Tyr1356). During cellular processes, substrate proteins undergo phosphorylation reactions leading to their polymerization and abnormal activation. This results in changes in cell proliferation, survival, apoptosis, invasion, migration, and blood vessel formation. Reactive ligands with high bioavailability bind to their highly expressed active receptors, becoming a common mechanism for antitumor drug targeting action. This adaptive mechanism is particularly important in tumor tissues characterized by hypoxia and inflammation. Many other RTKs have also been shown to play important roles in the TME, including the Eph receptor family, VEGFR and PDGFR.^139^, ^140^, ^141^

### 4.6 RTKs as stem cell markers

It is well established that a subset of cancer cells known as cancer stem cells (CSCs) contributes to tumor heterogeneity, metastasis, and resistance to treatment.^142^ RTKs play a crucial role in maintaining the characteristics of CSCs, such as self‐renewal capacity, viability, invasiveness, and tumorigenicity. Several RTKs have been identified to be involved in CSC maintenance, including Eph receptor,^143^ MET,^144^ EGFR,^145^ and others. Taking MET as an example, it is primarily expressed in stem and progenitor cells in normal tissues, while its expression is absent in cells that possess the ability to differentiate. As a result, it is believed that MET can be used to identify the functional status of CSCs since it is widely expressed in certain cancers, which could serve as a marker for the proliferation of cells with stem or progenitor cell characteristics.^146^

MET has the ability to promote stem cell‐like functions that are crucial for tumor initiation, multiplication, regeneration, and dissemination. This activity occurs regardless of whether MET is oncogenically activated or has prosurvival activity. In pancreatic cancer, MET serves as both a marker and therapeutic target for CSCs,^147^ Additionally, the interaction between HGF/MET and tumor‐stromal cells helps to maintain the preferred glucose metabolism of pancreatic tumor stem cells.^148^

The interaction between HGF and MET can cause abnormal expression of the wnt/β‐catenin pathway, leading to metastasis in colon cancer.^149^ Meanwhile, in HCC, the MET/FRA1/HEY1 cascade reaction, which is activated in CAFs, plays a vital role in regulating self‐renewal in CSCs.^150^ By encouraging cancer cells to migrate through the epithelial‐mesenchymal transition (EMT) process, activation of the MET pathway plays a critical role in boosting the preservation of CSCs within tumors and enhancing metastasis.^151^, ^152^, ^153^

## 5 RTKs EXPRESSION IN TUMORS

According to the most recent figures available, the malignancies with the highest number of new cases worldwide are prostate cancer, lung cancer, and CRC for men. For women, the highest number of new cases worldwide are breast cancer, lung cancer, and CRC. Therefore, this overview will focus on the expression of RTK in breast, prostate, lung, and colorectal tumors.

### 5.1 Breast cancer

Breast cancer is the leading cause of morbidity and mortality among women worldwide. The development of breast cancer is attributed to the deregulation of multiple signaling pathways in the breast epithelial cells. Activation of various signaling cascades by growth factors and chemokines occurs in the TME, ultimately promoting tumor growth. VEGFRs, EGFRs, FGFRs, and PDGFRs are present in different malignancies, including breast cancer. Elevated levels of RTKs have been associated with increased aggressiveness of breast cancer and lower overall and disease‐free survival (DFS).^154^

The significance of angiogenesis in the development of breast tumors has been extensively studied. VEGF, a potent proangiogenic factor, stimulates both lymphangiogenesis and tumor angiogenesis by binding to three different types of VEGFRs.^155^, ^156^, ^157^ The expression of VEGFR1 was found to be significantly higher in breast tumor tissues, regardless of lymph node metastasis, compared with benign tumors or surrounding healthy tissues.^158^ VEGF induces the production of VEGFR2, which then activates the JAK2/STAT3 signaling pathway, leading to the upregulation of Myc and Sox2 expression. In triple‐negative breast cancer (TNBC), the autocrine loop formed by the VEGF/VEGFR2 axis involving STAT3, Myc, and Sox2 contributes to the enhancement of the tumor stem cell‐like phenotype.^159^ Neolymphatic vascular development plays a critical role in the spread of cancer cells and the formation of distant metastases. Therefore, targeting the growth of new lymphatic vessels has emerged as a potential therapeutic strategy for breast cancer. However, the progress in developing antilymphangiogenic treatments for different types of cancer has been impeded by the lack of suitable markers to accurately assess lymphatic vessels and lymphatic metastases.^160^ VEGFR3 is an RTK that is expressed on lymphatic endothelial cells and plays a crucial role in the process of lymphangiogenesis.^55^ The VEGF‐C/VEGFR3 axis promotes lymphangiogenesis and the expression of VEGFR3. In the context of postnatal breast cancer, COX‐2 facilitates the metastasis of lymph nodes by promoting lymphangiogenesis and upregulating VEGFR3 expression.^161^, ^162^ Galectin‐8‐mediated crosstalk between the VEGF‐C, podoplanin, and integrin pathways plays a crucial role in lymphangiogenesis, and the presence of VEGFR3 is vital for this process.^163^ Targeting lymphangiogenesis by using anti‐VEGFR3 could potentially extend patient survival and decrease the dissemination of malignant cells.

Breast cancers with higher levels of EGFR overexpression have been associated with increased aggressiveness and poorer clinical outcomes. EGFR activates numerous downstream signaling molecules that promote cell proliferation, survival, and tumor progression. In a study conducted on 220 breast cancer patients, immunohistochemistry was employed to examine the expression of EGFR1, HER2, EGFR3, and EGFR4. The study revealed that EGFR1 was overexpressed in 16.4% of the tissues, HER2 in 22.8% of the tissues, EGFR3 in 17.5% of the tissues, and EGFR4 in 11.9% of the tissues.^164^ Upon binding to ligands, EGFR activates several downstream signaling molecules, including RAS, PI3K, phospholipase C‐γ (PLC‐γ), and JAK. This activation leads to cell survival, cell growth, and tumor progression.^145^, ^165^, ^166^ Inflammatory breast cancer (IBC) is characterized by NF‐κB activation, which causes endoplasmic reticulum (ER) downregulation, overexpression of EGFR and ErbB2, and hyperactivation of MAPK.^167^ Additionally, Nodal signaling, regulated by the EGFR/cyclooxygenase‐2 (COX‐2) axis, promotes the CSC phenotype and enhances the invasive capacity of IBC cells by inducing EMT.^168^ Animal models have shown that TAMs activation in cancer cells leads to STAT3‐mediated Sox2 expression, resulting in an increase in the number of tumor stem cells and metastasis in a mouse breast cancer model.^169^

Upon ligand stimulation, members of the FGFR family can activate pathways such as RAS/MAPK and PI3K/AKT, which play crucial roles in cell survival, proliferation, apoptosis, and migration. Notably, FGFR1 gene amplification has been observed in metastatic lobular breast cancer, ER^+^ breast cancer, and HER2^−^ breast cancer.^170^, ^171^ Furthermore, the expression of FGFR2 and FGFR3 is associated with the progression of ER+ breast cancer.^172^ FGFR4 and ErbB2 collaboratively regulate cyclin D1 expression, thereby promoting cell proliferation in breast cancer.^173^ These findings highlight the interconnectedness of FGFRs with other RTKs and their implications in resistance mechanisms. Consequently, targeting FGFRs holds promise as a therapeutic approach for breast cancer.

PDGFRs, which include both PDGFR‐α and PDGFR‐β family members, are found to be highly expressed in breast tumors and mesenchymal cells. Numerous studies have indicated that PDGFR expression is correlated with a poor prognosis in breast cancer patients, suggesting its prognostic and predictive potential.^174^, ^175^, ^176^ Moreover, PDGFRs have also been observed in reactive connective tissue mesenchyme, implying their potential role in tumor–mesenchymal interactions alongside their direct influence on cancer cells.^175^, ^176^, ^177^ Therefore, targeting PDGF/PDGFR in the TME holds promise as a therapeutic strategy for the treatment of TNBC.

### 5.2 Prostate cancer

FGF plays a complex role in the physiopathology of the prostate, from fundamental roles throughout embryonic development to regulation of tumor transformation. Maintaining a healthy FGF/FGFR signaling axis in the adult prostate is crucial for maintaining organ homeostasis and function. Disruption of this signaling axis can lead to prostatic hyperplasia and may promote cancer development and metastasis. Studies on FGF/FGFR can help overcome the challenges of treating prostate tumors.

While FGFR‐activating mutations are involved in various human tumors, prostate cancer commonly exhibits ectopic/altered levels of FGFR expression. Malignant prostate tumors frequently show ectopic expression of FGFR1, which triggers an autocrine loop due to aberrantly expressed FGFs.^178^, ^179^, ^180^ This autocrine loop promotes cancer cell autopoiesis, stimulates cell proliferation and migration, and prevents cancer cell death.^181^ Furthermore, ectopic FGFR1 has the ability to reorganize cellular energy metabolism in prostate cancer cells^182^ and stimulate inflammatory responses by activating NF‐κB signaling.^183^ As prostate cancer progresses and differentiation decreases, FGFR1 expression increases.^184^ Various in vivo studies have shown that FGFR2 expression either suppresses prostate cancer growth and progression or has no effect, in contrast to the significant role played by FGFR1 in this process. In fact, the absence of FGFR2 expression affects the mesenchymal‐epithelial signaling axis and is associated with prostate cancer development.^178^ The restoration of FGFR2 in human prostate cancer cells enhances their sensitivity to chemotherapeutic agents.^185^ Therefore, in mouse models of prostate cancer cells, chemically induced FGFR1 dimerization/activation leads to rapid tumor growth, while inducible FGFR2 expression slows down tumor growth. Some low‐grade prostate tumors exhibit somatic mutations in the FGFR3 gene, suggesting its involvement in FGF signaling in prostate cancer.^186^ FGFR4 is expressed in tubular epithelial cells of penile intraepithelial neoplasia and prostate cancer tissues.^187^

Bone metastases in patients with advanced prostate cancer are a life‐threatening condition. The development of bone metastases depends on the complex interactions among prostate cancer cells, osteoblasts, osteoclasts, and the bone matrix.^188^ Prostate cancer cells release growth factors and cytokines that stimulate bone cells.^189^, ^190^ These factors prompt osteoblasts to multiply and deposit new bone tissue. Consequently, osteoblasts and bone‐derived substances are released, which further stimulate the proliferation of cancer cells and activate osteoblasts. Throughout the osteogenic spectrum, FGF plays a critical role in regulating bone formation.^191^, ^192^ The interaction between FGFRs and FGF2 in osteoblasts triggers the activation of the p42/44 MAPK and PKC signaling pathways. PKC activation leads to increased expression of RUNX2, a transcription factor essential for osteoblast development.

The molecular mechanisms underlying FGF/FGFR‐mediated modulation of angiogenesis in prostate cancer were investigated through various preclinical studies conducted in different experimental settings. One such study involved in vitro and in vivo tests, which demonstrated that downregulating FGF2 in TRAMP‐C2 cells by overexpressing the tumor suppressor SEF‐b resulted in decreased angiogenesis.^193^ Additionally, activating FGFR1 conditionally in mouse prostate epithelium induced angiogenesis by upregulating HIF‐1α, VEGF, and angiopoietin‐2.^194^ These reports provide a theoretical basis for utilizing the suppression of the FGF/FGFR system as a combined antitumor/antiangiogenic therapy, with potential clinical implications for prostate cancer treatment. Overall, these findings suggest that the FGF/FGFR system plays a significant role in neointima formation in prostate cancer.^195^

Lymphangiogenesis plays a crucial role in the metastasis of tumors to both regional lymph nodes and distant organs.^196^, ^197^ Its association with tumor progression and unfavorable prognosis in prostate cancer has been well established.^198^, ^199^, ^200^ Studies have revealed that FGF2, derived from prostate tumor cells, can potentially stimulate lymphocytes by activating the FGFR1/AKT/mTOR/p70S6 kinase pathway.^201^

### 5.3 Lung cancer

Following the 2015 World Health Organization classification, lung cancer is now commonly referred to as NSCLC, while SCLC is included in the new category of neuroendocrine tumors.^202^ NSCLC can be further divided into three distinct subgroups: squamous cell carcinoma, adenocarcinoma, and large cell carcinoma. The majority of patients with NSCLC are diagnosed at an advanced stage, and their median survival time after diagnosis is typically less than one year.^203^

The identification of the echinoderm microtubule‐associated protein‐like protein 4 (*EML4*)‐*ALK* fusion gene in a group of NSCLC patients was first reported in 2007. Oncogenic *ALK* rearrangements result in ALK overexpression and constant activation, independent of ligands, by connecting the intact kinase structural domain of ALK to the Amino‐(N‐)terminal region of its partner. While other fusion partners of ALK have been found, EML4–ALK is the most prevalent type.^204^ *ALK* rearrangements occur in 3−7% of NSCLC, primarily in the lung adenocarcinoma (LUAD) subtype.^205^ Although ALK‐positive NSCLC patients represent a small percentage of all NSCLC cases, they contribute significantly to the overall number of lung cancer cases worldwide. Studies have demonstrated the oncogenic potential of *EML4–ALK* in the development of lung cancer in mice. By overexpressing *EML4–ALK* in lung type II alveolar cells, researchers observed rapid tumor growth with LUAD characteristics.^206^, ^207^ Another study induced *EML4–ALK* rearrangements in vivo using CRISPR/Cas9 gene editing, which also led to the formation of lung tumors.^208^ These animal models are valuable as they exhibit sensitivity to ALK inhibition, allowing for the exploration of the mechanisms underlying *EML4–ALK*‐induced lung cancer and the evaluation of ALK‐targeted therapy's effectiveness.

The dysregulation of the kinase activity of the EGFR protein can occur due to various oncogenic processes, including *EGFR* gene mutations, increased gene copy number, and overexpression of the EGFR protein.^209^ The EGFR family of receptors and ligands plays a crucial role in the complex interactions between tumor cells and the TME. Additionally, EGFR may interact with the integrin pathway and activate matrix metalloproteinases, which can modify cell adhesion, stimulate cell motility and invasion, and facilitate metastasis.^210^ Among the originally reported tumors with *EGFR* mutations, three were classified as fine bronchioloalveolar carcinomas, suggesting a higher prevalence of *EGFR* mutations in adenocarcinomas of the lung with features of fine bronchioloalveolar carcinoma.^211^ While some studies have yielded similar findings, others have not definitively established a link between *EGFR* mutations and specific subtypes of adenocarcinoma.^212^, ^213^, ^214^, ^215^ Furthermore, investigations have shown the presence of *EGFR* mutations in healthy fine bronchial epithelium alongside mutation‐positive adenocarcinomas, indicating that, in certain cases, *EGFR* mutations may occur early in the development of LUAD.^216^

MET, a receptor protein mainly found in epithelial cells, plays a crucial role in embryogenesis, tumor growth, and metastasis.^217^ The *MET* proto‐oncogenes encode these receptor proteins, which regulate genetic programs associated with cell proliferation and invasion of the extracellular matrix. In NSCLC, primary *MET* gene amplification has been observed in 1−5% of cases. However, it is more significant as it is also a mechanism of acquired resistance to EGFR‐TKIs, observed in 5−20% of cases.^133^ Another way MET activation occurs in NSCLC is through point mutations, particularly the elimination of exon 14, which is observed in 2−4% of NSCLCs and is more common in lung sarcomatoid carcinomas.^73^, ^218^ These mutations have also been linked to other malignancies such as gastric cancer (7%) or CRC (0–9%).^72^ It is worth noting that *MET* alteration is a recurring and actionable mechanism of resistance in ALK‐positive lung cancer.^219^ Also, KRAS acquired bypass resistance mechanisms include *MET* amplification.^220^

### 5.4 Colorectal cancer

**FIGURE 3:**
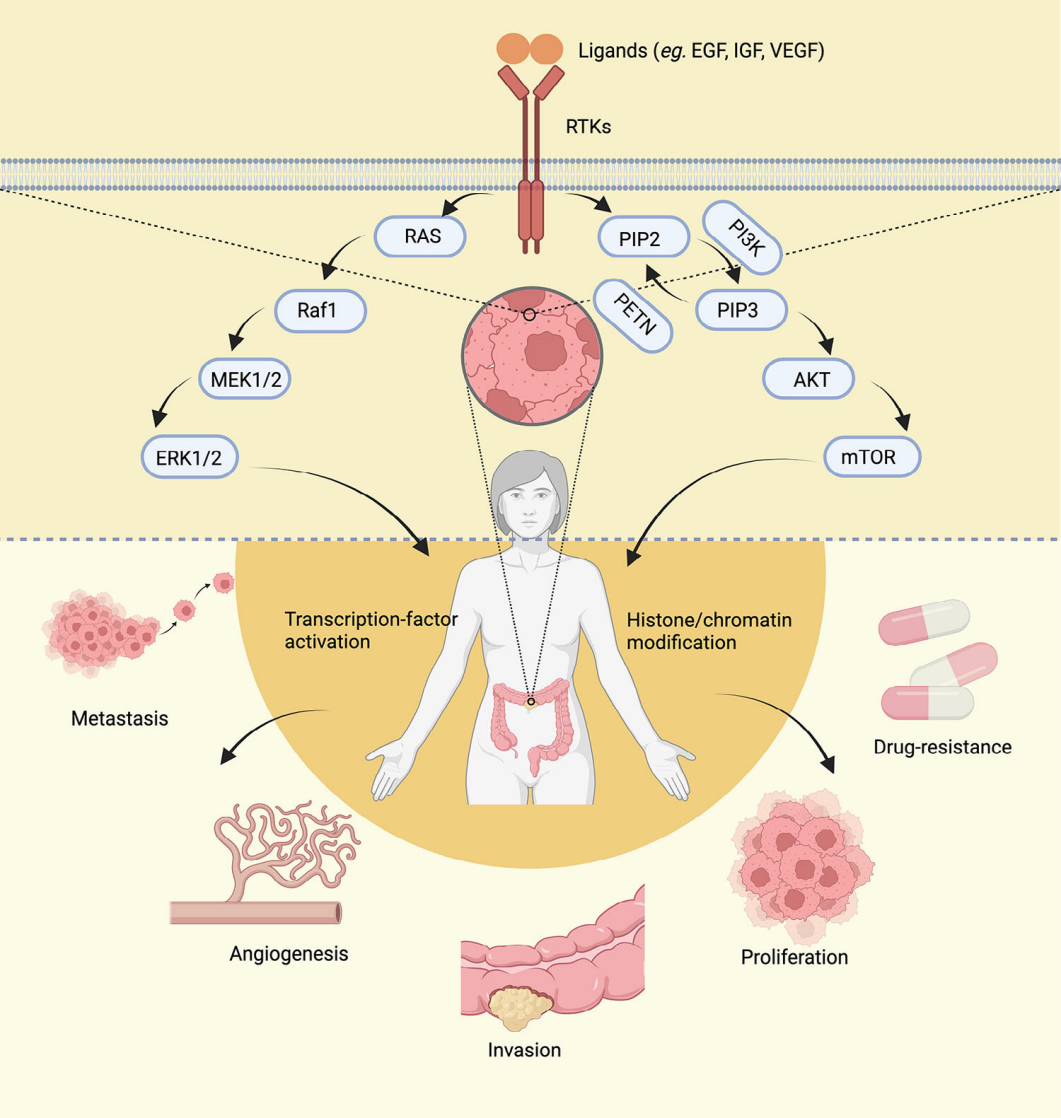
RTKs play a crucial role in the development of CRC. RTKs affect transcription‐factor activation and histone/chromatin modification by activating downstream pathways such as MAPK/ERK and PI3K/AKT, which in turn cause proliferation, resistance, metastasis, angiogenesis, and invasion of colorectal tumors. Created with BioRender.com.

Bowel, colon, and rectal cancers are commonly classified as CRC due to their shared characteristics. Metastasis is the primary factor contributing to cancer‐related deaths in patients with CRC. The majority of colon cancer cases originate from small, benign adenomatous polyps that eventually become malignant. While conventional chemotherapy has significant benefits in cancer treatment, the development of secondary resistance and nonspecific toxicity to rapidly dividing cells pose major challenges in achieving optimal outcomes. However, the field of molecular oncology has made remarkable advancements, enabling the development of highly selective medications that induce cancer cell death by targeting specific genes or proteins involved in cell proliferation or antiapoptosis.^221^ RTKs play a crucial role in the development of CRC (Figure 3).

In CRC cell lines, the *PDGFR* gene is frequently found to be overexpressed or mutated.^222^ The overexpression of PDGFR has been identified as a useful biomarker for detecting and managing CRC. It has been linked to angiogenesis, invasion, metastasis, poor survival, and resistance to targeted therapies in CRC patients.^223^

All four FGFRs and their corresponding ligands are expressed in CRC.^224^ Among them, FGFR1 is frequently overexpressed in CRC patients and is associated with aggressive clinical behavior.^225^ Additionally, FGFR2 regulates CRC cell migration, invasion, and growth, playing a crucial role in cancer progression.

NTRK1, NTRK2, and NTRK3 receptors are activated by NGF, BDNF, and neurotrophic factor‐3, respectively. These receptors play a role in the proliferative and migratory processes in the nervous system.^22^

ROR1 has been identified as both a prognostic marker and a potential therapeutic target for CRC. It is frequently overexpressed in CRC cells compared with healthy tissues and shows a positive correlation with clinical stage and lymph node metastases.^226^ The overexpression of ROR1 in CRC cells relative to surrounding normal tissues suggests its potential as an indicator of prognosis and a target for therapy in CRC.

High levels of HGF have been recognized as a significant prognostic marker in CRC. This is because HGF has the ability to promote the proliferation, motility, adhesion, and invasion of CRC cells, and is closely associated with the development, progression, and spread of CRC.^227^ In CRC, elevated levels of HGF often coincide with the overexpression of the MET receptor. The MET proteins, in turn, activate various proteins such as survivin, the X‐linked inhibitor of apoptosis protein, and other inhibitory apoptotic proteins through the AKT pathway, leading to tumor infiltration and distant metastasis.^228^

The AXL tyrosine kinase receptor is highly expressed in CRC^229^, ^230^ and is involved in various processes such as epithelial‐to‐mesenchymal transition, tumor angiogenesis, resistance to chemotherapy and targeted therapies, and suppression of the antitumor immune response.^230^ AXL is considered a potential therapeutic target for the treatment of CRC, especially in cases where adjuvant therapy targeting EGFR/VEGF has been ineffective.^231^

During the early stages of malignant transformation in CRC, the expression of EphA1, EphA2, EphB1, EphB2, and EphB4 is increased, suggesting their potential involvement in tumor invasion and metastasis.^232^ However, as CRC progresses, the expression of Eph proteins generally diminishes and eventually disappears in advanced CRC. Nonetheless, the expression of Eph proteins has been acknowledged as a potentially valuable marker in identifying individuals with CRC.^232^

In CRC, RET is considered a tumor suppressor kinase.^233^ The inactivation of the RET gene, due to abnormal methylation or mutation, may contribute to the progression of colonic adenomas into cancer.^233^, ^234^

Besides the mentioned RTKs, several other receptors including VEGFR,^235^, ^236^ EGFR,^227^, ^237^ STYK1,^238^ ALK,^239^, ^240^ LMTK3,^241^ TIE2,^242^ ROS1,^239^, ^240^ IGF1R,^20^ TYRO3,^243^ and MER^243^ have also been found to have prognostic significance in CRC.

## 6 ADVANCES IN RTKs TARGETED ANTICANCER THERAPY

Traditional radiotherapy and chemotherapy treatments for tumors are often associated with significant side effects as they are unable to distinguish between tumor cells and normal cells in the body. To address this issue, researchers have focused on suppressing cell signaling pathways as a potential approach for developing novel anticancer medications.^244^ Encouraging progress has been made in this area. For instance, in 1998, the United States Food and Drug Administration (US FDA) approved Genetech's first humanized monoclonal antibody called Herceptin, which specifically targets HER2, for the treatment of metastatic breast cancer.^245^ In 2001, Gleevec, the first small molecule BCR–ABL TKI, was introduced to the market as a treatment for chronic myelogenous leukemia (CML). This marked a significant milestone in the development of a new generation of antitumor drugs.^104^

The first generation of targeted TKIs includes imatinib,^246^ gefitinib,^247^ erlotinib,^247^ Icotinib,^248^ sorafenib,^249^ sunitinib,^250^ and crizotinib.^251^ However, resistance to these TKIs, kinase pathway crossover, and compensatory mechanisms have led to the development of second‐generation TKIs with more diverse targets. Examples of second‐generation TKIs are lapatinib,^252^ axitinib,^253^ afatinib,^254^ dacomitinib,^255^ and ceritinib.^256^ Third‐generation TKIs, such as osimertinib, loratinib, and others, are more selective, have superior therapeutic effects, and are less toxic compared with the first two generations.^257^, ^258^ RTKs are important signaling targets in vivo, and thus there are numerous therapeutic drugs specifically designed to target them. As of 2023, the US FDA has approved 40 TKIs worldwide for marketing.^259^ The US FDA‐approved TKIs for RTKs are listed in Table 2.

**TABLE 2:** US FDA‐approved TKIs for RTKs.

Drug	Code	Company	Trade name	Year approved	Primary targets	Therapeutic indications
Gefitinib	ZD1839	AstraZeneca	Iressa	2003	EGFR	NSCLC with exon 19 deletions or exon 21 substitutions
Erlotinib	OSI‐774	Genentech	Tarceva	2004	EGFR	NSCLC, pancreatic cancer
Sorafenib	BAY 43−9006	Bayer	Nexavar	2005	VEGFR1/2/3	HCC, RCC, differentiated thyroid cancer
Sunitinib	SU11248	Pfizer	Sutent	2006	VEGFR2	GIST, pancreatic neuroendocrine tumors, RCC
Lapatinib	GW572016	GSK	Tykerb	2007	EGFR, ErbB2/ HER2	HER2‐positive breast cancer
Pazopanib	GW786034	GSK	Votrient	2009	VEGFR1/2/3	RCC, soft tissue sarcomas
Vandetanib	ZD6474	Sanofi	Zactima	2011	VEGFR2	Medullary thyroid cancer
Crizotinib	PF 2341066	Pfizer	Xalkori	2011	ALK, ROS1	ALK or ROS1‐postive NSCLC, inflammatory myofibroblastic tumors, anaplastic large cell lymphoma
Cabozantinib	BMS‐907351	Exelixis	Cometriq	2012	RET, VEGFR2	Medullary thyroid cancer, RCC, HCC
Axitinib	AG‐013736	Pfizer	Inlyta	2012	VEGFR1/2/3	RCC
Regorafenib	BAY 73−4506	Bayer	Stivarga	2012	VEGFR1/2/3	Colorectal cancer, GIST, HCC
Afatinib	BIBW 2992	Boehringer Ingelheim	Tovok	2013	ErbB1/2/4	NSCLC, squamous NSCLC
Nintedanib	BIBF‐1120	Boehringer	Ingelheim Vargatef	2014	FGFR1/2/3	Idiopathic pulmonary fibrosis
Ceritinib	LDK378	Novartis	Zykadia	2014	ALK	ALK‐positive NSCLC resistant to crizotinib
Alectinib	CH5424802	Roche	Alecensa	2015	ALK, RET	ALK‐positive NSCLC
Lenvatinib	AK175809	Easai Co.	Lenvima	2015	VEGFR, RET	Differentiated thyroid cancer
Osimertinib	AZD‐9292	AstraZeneca	Tagrisso	2015	EGFR, T970M	NSCLC with exon 19 deletions or exon 21 substitutions
Brigatinib	AP 26113	Ariad Pharm	Alunbrig	2017	ALK	ALK‐positive NSCLC
Neratinib	HKI‐272	Puma Biotech	Nerlynx	2017	ErbB2/HER2	HER2‐positive breast cancer
Midostaurin	Novartis	Novartis	Rydapt	2017	Flt3	AML, mastocytosis, mast cell leukemia
Lorlatinib	PF‐06463922	Pfizer	Lorbrena	2018	ALK	ALK‐positive NSCLC
Fostamatinib	R788	Rigel Pharma.	Tavalisse	2018	Syk	Chronic immune thrombocytopenia
Dacomitinib	PF‐00299804	Pfizer	Visimpro	2018	EGFR	EGFR‐mutant NSCLC
Larotrectinib	LOXO‐101	Bayer	Vitrakvi	2018	TRKA/B/C	Solid tumors with NTRK fusion proteins
Gilteritinib	ASP2215	Astellas Pharma	Xospata	2018	Flt3	AML with FLT3 mutations
R406 active metabolite of fostamatinib		Rigel Pharma.		2018	Syk	Chronic immune thrombocytopenia
Erdafitinib	JNJ‐42756493	Jansen Pharm	Balversa	2019	FGFR1/2/3/4	Urothelial bladder cancer
Entrectinib	RXDX‐101	Ignyta, Inc.	Ignyta	2019	TRKA/B/C, ROS1	Urothelial bladder cancer
Pexidartinib	PLX3397	Plexxikon Inc	Turalio	2019	CSF1R	Tenosynovial giant cell tumors
Avapritinib	BLU285	Blueprint Medicines	Ayvakit	2020	PDGFRα	GIST with PDGFRα exon 18 mutations
Pralsetinib	Blu‐667	Blueprint Medicines	Gavreto	2020	RET	RET‐fusion (i) NSCLC, (ii) medullary thyroid cancer, (iii) differentiated thyroid cancer
Pemigatinib	INCB054828	Incyte Corp.	Pemazyre	2020	FGFR2	Cholangiocarcinoma with FGFR2 fusions or other rearrangements
Ripretinib	DCC‐2618	Deciphera Pharma.	Qinlock	2020	Kit, PDGFRα	Fourth‐line treatment for GIST
Selpercatinib	CEGM9YBNG	Lilly	Retevmo	2020	RET	RET fusion NSCLC and thyroid cancers and RET mutant medullary thyroid cancer
Capmatinib	INC‐280	Novartis	Tabrecta	2020	MET	NSCLC with MET exon 14 skipping
Tucatinib	ONT‐380	Seattle Genetics	Tukysa	2020	ErbB2/HER2	Combination second‐line treatment for HER2‐positive breast cancer
Mobocertinib	TAK‐788	Takeda Pharm.	Exkivity	2021	EGFR	NSCLC with EGFR‐positive exon 20 insertions
Tivozanib	AV951	AVEO Pharma	Fotvida	2021	VEGFR2	Third‐line treatment of RCC
Tepotinib	EMD 1214063	EMD Serono Inc.	Tepmetko	2021	MET	NSCLC with MET mutations
Infigratinib	BGJ 398	QED Therapeutics	Truseltiq	2021	FGFR2	Cholangiocarcinomas with FGFR2 fusions or other rearrangements
Futibatinib	TAS_120	Tiaho Pharma	Lytgobi	2022	FGFR2	Bile duct cancers (cholangiocarcinomas) with FGFR2 fusions or other rearrangements

Abbreviations: AML, acute myelogenous leukemia; ErbB2/HER2, human epidermal growth factor receptor‐2; GIST, gastrointestinal stromal tumor; HCC, hepatocellular carcinoma; NSCLC, non‐small cell lung cancer; RCC, renal cell carcinoma; SLL, small lym.

**FIGURE 4:**
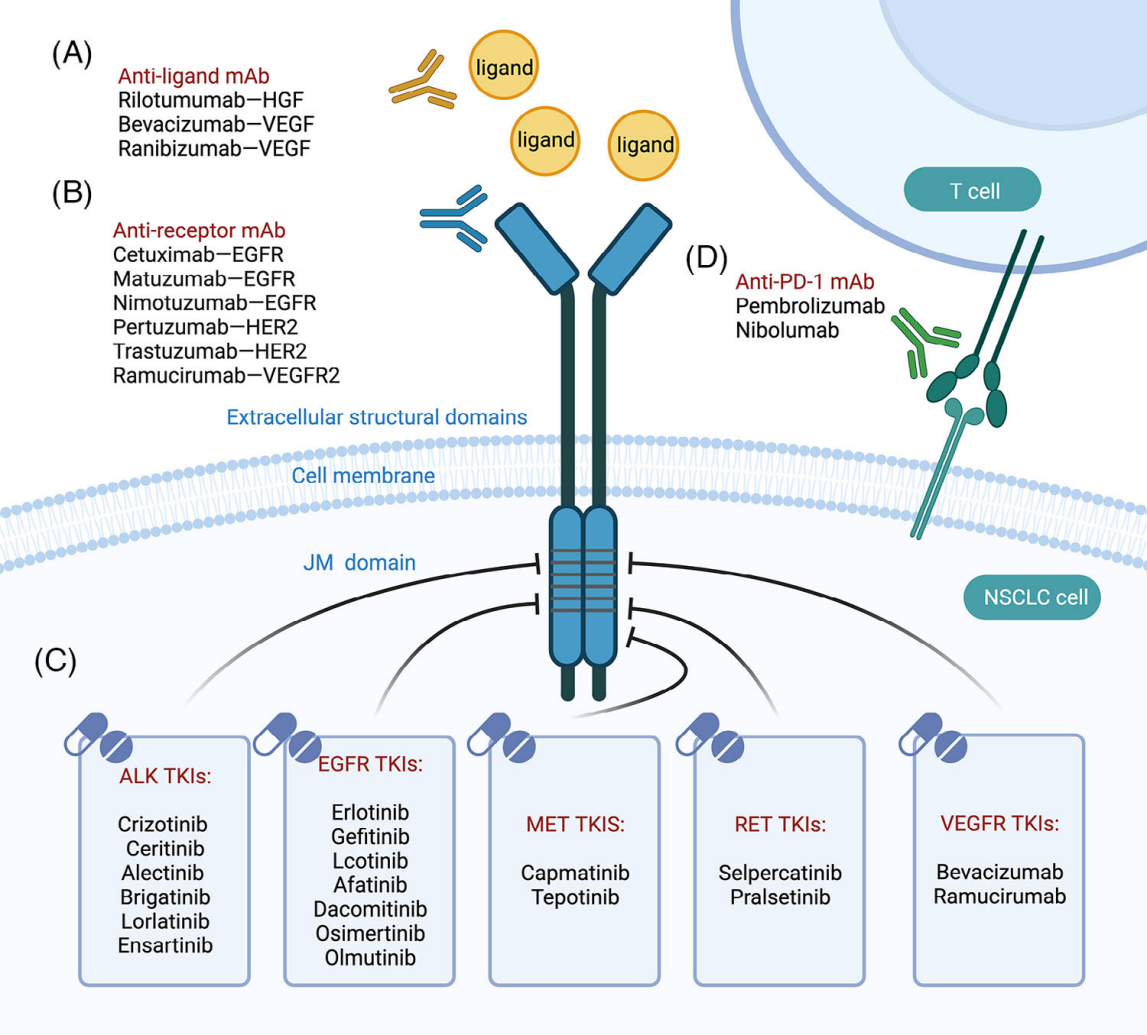
Targeted therapeutic modalities in NSCLC. (A) Anti‐ligand antibodies include rilotumumab, bevacizumab, and ficlatuzumab. (B) Large‐molecule biologics target the RKTs protein found on the surface of tumor cells and prevent it from binding to ligands. Some examples of anti‐receptor antibodies include cetuximab, matuzumab, nimotuzumab, pertuzumab, trastuzumab, and ramucirumab. (C) Specific tyrosine kinase inhibitors and multitarget tyrosine kinase inhibitors are small molecules that act on the juxtamembrane domain of RTKs. (D) Anti‐PD1 antibodies antitumor by immune means, including pembrolizumab and nivolumab. Created with BioRender.com.

Drugs targeting tyrosine kinases include antibody‐based drugs and TKIs. For example, in NSCLC, targeted therapies can be classified into five categories: multitargeted tyrosine kinase inhibitors (small molecules), specific tyrosine kinase inhibitors (small molecules), anti‐receptor antibodies, anti‐ligand antibodies, and immunotherapies. These drugs exert their effects by acting on different sites to inhibit signaling pathways (Figure 4).

### 6.1 Multitarget tyrosine kinase inhibitors (small molecules)

Crizotinib is an ATP‐competitive multitarget protein kinase inhibitor that targets Met, ALK, and ROS1. It has shown promise in treating patients with ALK‐positive NSCLC. However, patients treated with crizotinib typically develop resistance within a year or two, and there is a higher incidence of central nervous system (CNS) progression or metastasis in ALK‐positive NSCLC patients who receive crizotinib as a first‐line treatment. As a result, second‐generation ALK inhibitors such as ceritinib, alectinib, brigatinib, and ensartinib, along with the third‐generation lorlatinib, have emerged as alternatives after crizotinib resistance.^260^

Loratinib, a reversible and potent third‐generation tyrosine kinase inhibitor, exhibits high selectivity by targeting ALK and ROS1. Notably, it has excellent penetration into the CNS and has shown effectiveness in treating patients with intracranial metastases after previous treatment with second‐generation ALK inhibitors.^261^ Several studies have demonstrated that patients treated with lorlatinib experience significantly longer progression‐free survival and a higher occurrence of intracranial reactions compared with those treated with crizotinib.^262^, ^263^

Cabozantinib, a broad‐spectrum anticancer medication, has the ability to inhibit multiple targets including MET, VEGFR1/2/3, RET, KIT, FLT3, AXL, NTRK, ROS1, and others. Currently, it is considered as the first‐line treatment for kidney cancer. Moreover, cabozantinib has shown remarkable efficacy in treating various other cancers such as thyroid cancer, liver cancer, soft tissue sarcoma, NSCLC, prostate cancer, breast cancer, ovarian cancer, and bowel cancer. In RCC, the combination of nivolumab and cabozantinib has demonstrated positive patient benefits.^264^

### 6.2 Specific tyrosine kinase inhibitors (small molecules)

Compared with first‐ and second‐generation TKIs, osimertinib is a third‐generation EGFR‐positive lung cancer targeted medication that significantly improves patients’ overall survival and progression‐free survival. In patients with EGFR‐mutated stage IB‐IIIA NSCLC, adjuvant osimertinib after full tumor excision dramatically increases DFS and reduces the risk of both local and distant recurrence.^265^ Osimertinib has also shown favorable patient benefit in combination with other drugs. Several trials have demonstrated that combining tepotinib and osimertinib could potentially overcome resistance caused by MET amplification and lead to a significant improvement in the objective remission rate for patients with MET‐amplified NSCLC.^260^, ^261^

Tepotinib, the first MET tyrosine kinase inhibitor approved for treating NSCLC, is currently being developed to treat solid tumors.^266^ The drug has demonstrated significant activity in subgroups of patients with varying ages, prior treatments, and brain metastases, while maintaining a reasonable safety profile and few treatment discontinuations.^267^

Tivantinib, a MET inhibitor, works by stabilizing the inactive conformation of the MET RTK. It prevents both constitutive and ligand‐mediated activation through an ATP‐independent binding mechanism. In patients with HCC who have failed sorafenib treatment, MET overexpression has been shown to be a negative prognostic indicator. In a second‐line therapy phase II study for advanced HCC patients, those in the MET high expression group who were given tivantinib showed almost double the PFS and OS compared with those who received a placebo. Furthermore, the combination of tivantinib and sorafenib also showed promising results.^268^

Savolitinib is a MET inhibitor that has been developed to treat metastatic NSCLC, PRCC, gastric cancer, and CRC.^269^ Preclinical data suggest that the combination of EGFR TKIs and MET TKIs may be a possible treatment option for EGFR mutation‐positive lung cancer with MET‐driven acquired resistance. In patients with advanced NSCLC whose disease has progressed after previous use of an EGFR TKI and who have *MET*‐amplification, the combination of savolitinib and osimertinib has shown promising antitumor activity with acceptable risk effects.^270^ In May 2020, the National Medical Products Administration of China accepted a new drug marketing application for the MET inhibitor savolitinib, intended for the treatment of NSCLC with *MET* exon 14 skipping. Savolitinib has been authorized as a novel medicine for both domestic and international markets.

SCC244 is a highly selective inhibitor of MET kinase that has demonstrated subnanomolar potency against MET kinase activity and excellent selectivity versus 312 other evaluated protein kinases. The administration of SCC244 has shown strong anticancer efficacy at well‐tolerated levels in xenografts of human tumor cell lines or NSCLC and HCC patient‐derived tumor tissue driven by MET abnormality. These findings provide a solid platform for the clinical examination of SCC244 in patients with malignancies that are altered along the MET pathway.^271^ A study demonstrated that when SCC244 and HAI‐2 were used together, they had a greater inhibitory effect on the proliferation of RCC cells compared with when either inhibitor was used alone.^272^

Capmatinib demonstrated significant antitumor activity in patients with advanced NSCLC characterized by *MET* exon 14 skipping mutations, particularly in those who had not received prior treatment. A clinical trial involving 364 patients evaluated the efficacy of capmatinib. Among patients with NSCLC and a *MET* exon 14 skipping mutation who had previously undergone one or two lines of therapy, an overall response rate of 41% (95% confidence interval [CI], 29–53) was observed. For patients who had not received any prior therapy, the overall response rate was 68% (95% CI, 48–84). The median duration of response was 9.7 months (95% CI, 5.6 to 13.0) for previously treated patients and 12.6 months (95% CI, 5.6 to could not be estimated) for treatment‐naïve patients. However, patients with *MET* amplification, who had undergone prior treatment and had a gene copy number of less than 10, exhibited limited efficacy with an overall response rate of 7−12%.^273^

### 6.3 Antireceptor antibodies

The US FDA authorized cetuximab, a chimeric murine human monoclonal antibody of the IgG1 subclass, in February 2004 for the treatment of advanced CRC. It was the first EGFR mAb to be approved. Early phase II clinical trials of cetuximab showed promising results.^274^ According to a randomized phase II trial, patients who were irinotecan‐refractory and received cetuximab + irinotecan had a median OS of 22.9 months (218 participants), while those who received irinotecan alone had a median OS of 10.8 months (111 subjects).^275^ However, the effectiveness and overall survival of cetuximab may be influenced by alterations in the *KRAS* gene. Currently, patients with *KRAS* wild‐type and EGFR‐positive metastatic CRC are treated with cetuximab and chemotherapy as their first‐line treatment.

SAIT301 has been found to downregulate MET and effectively inhibit the growth of tumors with low or no Cbl expression. It also has the ability to prevent the growth of tumors with the deletion of *MET* exon 14, where MET binds to Cbl.^276^ According to a study, SAIT301 utilizes a MET degradation pathway that is mediated by LRIG1 and independent of Cbl. This pathway avoids the effect that has been known to limit the effectiveness of MET antibodies.^276^ SAIT301 has significant implications in the diagnosis and treatment of nasopharyngeal carcinoma. The inhibitory effect of SAIT301 on MET significantly reduces the migration and invasion of HNE1 cells. Additionally, SAIT301 can also decrease the nonanchored growth of HGF‐induced HNE1 cell lines.^277^ A study found that the anticancer activity of SAIT301 was enhanced in *MET*‐amplified MKN45 cells when 69 genes were knocked down.^278^ Further analysis of these genes revealed that FGF receptor (FGFR) is a crucial regulator of the antiproliferative effects of MET‐targeted drugs. Integrin β3 is also a promising target for combination therapy with SAIT301. Gene expression analysis using the CCLE database suggests that FGFR and integrin β3 can be used as predictors of MET‐targeted therapy,^278^ offering a potential therapeutic solution to overcome the acquired and innate resistance to MET‐targeted drugs.

### 6.4 Antiligand antibodies

Bevacizumab is the first medication authorized for sale that targets tumor angiogenesis. It has been proven effective in treating various malignant tumors and is approved for multiple indications, including metastatic CRC, NSCLC, breast cancer, GBM, ovarian cancer, RCC, and cervical cancer. Its mechanism of action involves selectively targeting VEGF to exert antitumor effects.^279^ Combining chemotherapy with bevacizumab has shown benefits for patients. A study revealed that patients with refractory metastatic CRC who received FTD‐TPI + bevacizumab treatment had longer overall survival compared with those who received FTD‐TPI alone.^280^ Bevacizumab marked the beginning of a novel approach to anticancer therapy and remains the most widely used antiangiogenic treatment.

Rilotumumab is a monoclonal antibody that targets the HGF protein, preventing it from binding to MET. A study revealed that the effectiveness of rilotumumab is dependent on the dose given to patients with MET‐positive gastric cancer or cancer of the esophagogastric junction.^281^ However, another study found that inhibiting the MET pathway with rilotumumab did not improve the clinical prognosis of patients with MET‐positive gastric or gastroesophageal adenocarcinoma.^282^ Resistance is a common obstacle to effective cancer treatment, and acquired resistance is becoming a significant issue for targeted therapies. Rilolotuzumab resistance is acquired through unusual mechanisms, including dramatic HGF overproduction and misfolding, ER stress response signaling, and redirected vesicle trafficking.^283^

### 6.5 RTKs inhibitors in combination with immunotherapy

While some immunotherapies, including pericyte transfer (ACT) and immune checkpoint inhibitors, have shown promise in providing long‐lasting clinical responses, their efficacy is not consistent and only a portion of cancer patients can benefit from them. The inflammatory response in the TME is a significant factor contributing to tumor progression and poor prognosis. However, accurately profiling immune cells within the TME has been limited due to its highly heterogeneous and dynamic nature. Fortunately, recent advancements in single‐cell technologies such as single‐cell RNA sequencing (scRNA‐seq) and mass spectrometry flow cytometry have made it possible to systematically detect immune cells within the TME and gain new insights into their functional diversity.^284^

Patients with advanced *MET* 14 exon alterations in lung cancer show a long‐lasting response to MET inhibitors. A study reveals that a considerable proportion of lung cancers with *MET* exon 14 alterations express PD‐L1, but their median tumor mutation burden is lower than that of unselected NSCLCs.^285^ Although PD‐1 blocking sometimes elicits responses, its overall clinical effectiveness is limited.

The interaction between MET and TME has been linked to secondary gliomas. However, the effect of *MET* genes on primary gliomas, specifically GBM, and their ability to evade immunosurveillance checkpoints is not well understood. A recent study proposes that the MET/STAT4/PD‐L1 axis and tumor‐associated macrophages may contribute to glioma immune evasion and lead to poor prognosis in GBM cases. This finding suggests a potential clinical approach for targeted therapy combined with immunotherapy for primary GBM patients.^286^

Pancreatic cancer is a highly malignant tumor with a complex immune microenvironment. Current RTK targeting strategies have limited effectiveness in treating this cancer. However, recent research has identified MET as a pancreatic cancer‐specific RTK that is significantly associated with prognosis in both immunologically “hot” and “cold” pancreatic cancers. Studies have shown that MET is highly upregulated in pancreatic cancer tissues and positively correlated with *PD‐L1* levels. Additionally, elevated MET and PD‐L1 expression have been strongly associated with lymph node metastasis, tumor TNM staging, and overall survival in pancreatic cancer. MET can interact with PD‐L1 and help maintain its expression level in various ways. Lowering the expression of MET can increase the infiltration of lymphocytes into pancreatic tumors.^287^

To enhance the clinical benefits, targeted medicines can be combined with different inhibitors and immunotherapy for the treatment of cancers. Currently, several clinical trials are underway to investigate new targeted medications. The outcomes of some clinical trials for RTKs are summarized in Table 3.

**TABLE 3:** Summary of clinical trials of TKIs targeting RTKs.

Targets	Drugs	Cancer types	Phase	*N*	Principal outcome	Study
EGFR‐mutant	Osimertinib	NSCLC	II‐IIIA	682	Median follow‐up was 44.2 months (osimertinib) and 19.6 months (placebo); the DFS HR was 0.23; 4‐year DFS rate was 70% (osimertinib) and 29% (placebo). In the overall population, DFS HR was 0.27; 4‐year DFS rate was 73% (osimertinib) and 38% (placebo).	NCT02511106
HER2‐low	Trastuzumab Deruxtecan	Advanced breast cancer	III	557	Among all patients, the median PFS was 9.9 months in the trastuzumab deruxtecan group and 5.1 months in the physician's choice group (hazard ratio for disease progression or death, 0.50; *p* < 0.001), and median OS was 23.4 months and 16.8 months, respectively (hazard ratio for death, 0.64; *p* = 0.001).	NCT03734029
HER2‐mutant	Trastuzumab Deruxtecan	NSCLC	II	91	The median duration of follow‐up was 13.1 months. The median duration of response was 9.3 months. Median PFS was 8.2 months, and median OS was 17.8 months.	NCT03505710
FGFR2‐rearranged	Futibatinib	Intrahepatic cholangiocarcinoma	II	103	The median DOR was 9.7 months. At a median follow‐up of 17.1 months, the median PFS was 9.0 months and median OS was 21.7 months.	NCT02052778
MET Exon 14‐mutated or MET‐amplified	Capmatinib	NSCLC	II	364	OR was observed in 41% of 69 patients who had received one or two lines of therapy previously and in 68% of 28 patients who had not received treatment previously; the median DOR was 9.7 months and 12.6 months, respectively, and in 40% of those who had not received treatment previously.	NCT02414139
MET‐ or ROS1‐positive	Crizotinib	NSCLC	II	90	The ORR was 16% in the MET ≥6 copies cohort, 10.7% in the mutated, and 47.2% in the ROS‐1 cohort. The best ORR during treatment was 32% in the MET‐≥6 copies cohort, 36% in the MET‐mutated, and 69.4% in the ROS‐1‐translocation cohort.	NCT02034981
ALK‐positive	Lorlatinib or Crizotinib	NSCLC	III	296	The percentage of patients who were alive without disease progression at 12 months was 78% in the lorlatinib group and 39% in the crizotinib group. An objective response occurred in 76% of the patients in the lorlatinib group and 58% of those in the crizotinib group; among those with measurable brain metastases, 82% and 23%, respectively, had an intracranial response, and 71% of the patients who received lorlatinib had an intracranial complete response.	NCT03052608
RET‐altered	Selpercatinib	Thyroid cancers	I‐II	55	Patients with RET‐mutant medullary thyroid cancer who had previously received vandetanib, cabozantinib, or both, the percentage who had a response was 69%, and 1‐year PFS rate was 82%. In 88 patients with RET‐mutant medullary thyroid cancer who had not previously received vandetanib or cabozantinib, the percentage who had a response was 73%, and 1‐year PFS rate was 92%. In 19 patients with previously treated RET fusion‐positive thyroid cancer, the percentage who had a response was 79%, and 1‐year PFS rate was 64%.	NCT03157128

Abbreviations: DFS, disease‐free survival; DOR, duration of response; HR, hazard ratio; NSCLC, non‐small cell lung carcinoma; OR, overall response; ORR, objective response rate.; OS, overall survival; PFS, progression‐free survival.

*Source*: ClinicalTrials.gov Home—ClinicalTrials.gov.

## 7 CONCLUSION AND PERSPECTIVE

This review outlines the 20 classifications of RTKs and explains the mechanisms of their activation. Special attention is given to the dysregulation of RTKs, which can lead to tumorigenesis and cancer progression. The review focuses on the specific oncogenic effects of various RTKs in different types of tumors. The recognition of the significance of RTKs as therapeutic targets in tumor therapy highlights the crucial role of tyrosine kinase receptors in the progression of tumors. It also provides guidance for the development of new targeted therapies or immunotherapies for the treatment of cancer.

At present, several important questions in cancer immunity require attention. First, it is important to investigate the involvement of RTKs in interfering with cancer immunity. Although EGFR‐TKIs and ALK‐TKIs are not recommended to be used with PD‐1/PD‐L1 antibodies, the combination of other RTK inhibitors with immunotherapy may potentially enhance the effectiveness of antitumor therapy. Second, the metabolic reprogramming of cancer cells plays a significant role in cell variety, proliferation, invasion, and metastasis. However, the metabolic status of cancer cells with RTK activation or inhibition remains largely unknown. Targeting the reprogrammed metabolism of cancer cells could potentially improve the therapeutic effects of TKIs on cancer. Third, the remodeling of the TME by TKIs is not well understood, but it is crucial for understanding resistance to therapy. Further research on the underlying mechanisms of TME remodeling will provide valuable insights for cancer treatment.

Future research should focus on three main areas in order to better utilize RTKs inhibitors in the treatment of human cancers. These include (1) optimizing the inhibitors to more effectively target the various RTKs alterations found in different cancers, (2) refining patient classification and selecting appropriate treatment modalities based on specific RTKs alterations to improve treatment efficacy, and (3) determining the relationship between RTKs and various aspects of cancer such as prognosis, diagnosis, postoperative recurrence, survival, and treatment resistance. By addressing these areas, we can more precisely use RTKs for clinical diagnosis and treatment of cancer.

## AUTHOR CONTRIBUTION

Yongsheng Li conceived this review. Nan Zhang and Yongsheng Li wrote and revised the manuscript. All authors have read and approved the final manuscript.

## CONFLICT OF INTEREST STATEMENT

The authors declare no conflicts of interest.

## ETHICS STATEMENT

Not applicable.

## Data Availability

Not applicable.
